# The Foreign Journals: Anatomy and Physiology

**Published:** 1839-04

**Authors:** 


PART THIRD.

### Selections from the British and Foreign Journals.

### I. THE FOREIGN JOURNALS.

### ANATOMY AND PHYSIOLOGY.

*On the Fibres of the Spinal Marrow and Sympathetic Nerve in the Rana Esculenta.* By Dr. A. W. Volkmann, Professor of Physiology in Dorpat.

[This paper forms an interesting addition to the treatises which are becoming daily more numerous, on the structure and arrangement of the primitive elements of the nervous system. Its author was led to an investigation of these organs through his enquiries into the phenomena of reflex-motory functions. These phenomena prove that the spinal cord is something more than the aggregate of the nerves proceeding from it—that it possesses attributes somewhat allied to the "organ of the soul," and, consequently, would lead us to expect that its structure is more nearly allied to the brain than to the nerves. And this is corroborated by observation. The author is of opinion that as yet we cannot presume to decide upon the subject of structure even, although the best authorities on this point differ less in their descriptions than in the conclusions they have drawn from their observations. We give here the principal results of the Professor's researches; leaving it, and, indeed, recommending it, to those who feel sufficient interest in the subject to peruse the original.]

The primitive fibres of the medullary substance appear sometimes cylindrical, and sometimes varicose: both forms occasionally presenting themselves in the same preparation. It is probable that in the undisturbed state only one of these characters prevails: and, as it is less probable that an uniform cylinder would be produced out of a varicose fibre than *vice-versâ*, it may be assumed that the cylinder is the natural condition, and that it becomes converted into the irregular varicose fibre by means of the disturbance consequent upon preparation. The most recent investigations of Krause, Treviranus, E. H. Weber, and myself, have tended to confirm the opinion that the cylindrical form is not only the most prevalent (to say the least) in the nervous system, but that in some parts it is the universal character of its fibres. The following are the principal grounds which lead to this conviction.

1. E. H. Weber and myself found that in the valvula cerebelli of small animals the fibres are uniformly cylindrical; a circumstance of great importance, since this is the only part of the nervous system thin enough to allow the structure to be investigated without previous preparation.

2. All observations showing the cylindrical structure to prevail in cases where water has been employed in the preparation are inadmissible, since water possesses the property of converting the cylindrical into the varicose form. If this be tried (as Krause has shown) it will be found that the fibres become at the same time evidently shortened.

3. Whenever mechanical force is applied, the same objection may be made; and it is not requisite that the nervous matter should have been formally prepared to show this, for the mere tearing or separating a portion of this substance is sufficient to give rise to this change.

4. It is found that the greater the care with which these disturbing agents are avoided, the fewer will be the number of the varicose fibres; sometimes, indeed, they are entirely absent. The best way to examine nervous substance is to make use of albumen, serum, or saliva, as the moistening medium; and, in order to get a portion sufficiently fine for viewing under the microscope, rely more upon the gentle separation of larger pieces, by means of fine needles, than endeavour to separate a portion sufficiently delicate at once by using scissors. In this manner single fibres frequently become visible, and are generally of a cylindrical form.

5. It not unfrequently happens, however, that even where these objectionable forces are applied, some of the fibres—and often to a considerable extent—present the cylindrical character. This circumstance proves that, however true it may be that the contact of water and the use of mechanical force generally produce an alteration in form, yet it is not so universal as the opponents of Ehrenberg maintain.

These facts lead me to the conclusions—

1. That the fibres of the brain and spinal marrow have, for the most part, a cylindrical form.

2. That the varicose fibres are, in the majority of instances, the result of preparation.

3. But that the existence of varicose fibres in some parts cannot be absolutely denied. The fibres of the spinal marrow in the frog, as, probably, in all other vertebrated animals, take a tolerably parallel course in the longitudinal direction, without the least indication of transverse fibres or anastomoses, which the phenomena of reflex functions would almost have led us to expect. As circumstances establishing the analogy between the structure of the brain and spinal marrow to be greater than that between the latter and the nerves—may be adduced: 1. The extreme fineness of the primitive fibres, the average of which may be stated at 0·00015 inch, whilst the measurement of the primitive filaments of the ischiatic nerve lies between 0·00045 and 0·00070 inch. 2. The frequent appearance of the varicose form, which, although it may be attributable to the causes named, becomes, nevertheless, in so far, characteristic of these portions of the nervous system. And 3. The easy disruption in a transverse direction, taking place without the application of more force than is necessary to separate the filaments one from the other. The reverse of this is the case in the nerves themselves; a circumstance which may, perhaps, with justice be attributed to the investing neurilema of the nervous fibres.

There are also globules within the substance of the nervous mass: these are considered by Ehrenberg to be merely the remains of fibres; but I am of opinion that they form an essential part of the medullary substance; for they are frequently found to exceed by far the dimensions of the filaments, nor are they angular or irregular, as we should in that case expect them to be; besides which, their position amidst the fibres, and their being devoid of such irregular portions of filament as must sometimes be left attached to them, provided they were broken down fibres, seem to indicate that they have a separate existence.

The spinal cord of the frog exceeds by far the combined volume of the nerves arising from it. The author gives a careful measurement of each nerve at its origin, and, doubling the total for both sides of the body, gives the proportional diameter of the nerves to the spinal cord as 0·0817 inch to 0·1100 inch. This fact and the circumstance above named, that the fibres of the spinal cord are much finer than those of the nerves themselves, seem likewise to justify the inference that the filaments of the former are (say $\frac{2}{3}$) more numerous than those of the latter. This question assumes considerable interest when we reflect upon the probable function of these supernumerary filaments, particularly in making comparative reference to the brain, which, without doubt, possesses a vast preponderance of fibres over and above such as seem to be continued in the substance of the nerves. This consideration, too, appears conclusive in regard to the opinion that the spinal cord deserves to be considered as a central organ. If those filaments which Ehrenberg first observed (and I have frequently confirmed his statement) to pass from the spinal cord along the nerves given off from it, are subservient to motion and sensation, as we may reasonably suppose, what is the function of those which remain? The superfluous fibres of the brain may be supposed to be necessary to the formation of the organs of the mind; but to what are those of the spinal cord subservient? There is a remarkable circumstance connected with the spinal marrow of the frog. Posterior to the point at which the tenth spinal nerve is given off it retains half its original size, extending through the remaining vertebræ and the anterior third of the long caudal bone, and in this course gives rise to three nervous filaments, which are scarcely visible to the naked eye.

Here then is a portion of the cord which evidently belongs to the central organ, and does not stand in immediate relation to the peripheral distribution of its nerves. It would be desirable to ascertain whether the fibres of the spinal cord—as well those which are continued into its nerves as those peculiar to it as a nervous centre—are continuous with those of the cerebrum. It is satisfactorily proved that many motory functions, as, for instance, the act of swallowing, depend on the spinal cord, and not on the brain; and it is, therefore, in some measure probable that such fibres take their origin in the former and not in the sensorium.

The results of the author's investigation of the mode of origin of the spinal nerves are interesting, but require corroboration. He says "The ganglia, through which the union of both nerves (the sensitive and motor) is effected, do not belong so entirely to the sensitive root as in the mammalia. In the fourth spinal nerve, the ganglion appears to be equally divided between the two roots. In the fifth, although the motor branch passes by the side of the ganglion, still examination with the microscope convinces me that some of its filaments pass through it. The tenth nerve has only one root, which is furnished with a ganglion at its point of exit from the canal. The sympathetic nerve unites with the anterior branches of all the spinal nerves excepting the first, which performs the function of the hypoglossus. It forms a double ganglial cord, closely approximating in the cervical region, and diverging towards its abdominal end, so as at last to be connected with the spinal nerves by long and slender filaments."

The author here gives a minute account of the precise mode of union between the individual ganglia and the spinal nerves; of this, however, we must be content to give a summary.

1. The sympathetic nerve consists of two kinds of filaments. One set of these belongs peculiarly to it, and may be named sympathetic in the strictest sense; the other takes its origin in the spinal cord, and may be termed the medullary fibres of the sympathetic. It is universally found that those spinal nerves which unite with the sympathetic are much thicker towards their origin than in the contrary direction; therefore, since it is not at all probable that the central organs receive so large a number of sympathetic fibres, it remains to be assumed that they are the filaments which spring from the central organs, and serve to communicate the influence exercised by the brain and spinal marrow upon the organs of vegetative life.

2. The sympatheticus increases the dimensions of all the spinal nerves, except the first, in joining them in the direction of their peripheral extremity. The first nerve, corresponding to the hypoglossus, is distributed to the tongue, which organ likewise receives branches from the vagus nerve, and this contains a considerable proportion of sympathetic filaments.

3. It is probable, from the mode of union of the ganglia with the spinal nerves, that filaments from various parts of the latter pervade the ganglionic system throughout its whole extent.

4. The superior part of the spinal cord appears to have a greater share in supplying the medullary fibres of the ganglionic system than the lower. The superior connecting branches contain decidedly more of them than the inferior; and the same remark holds good with regard to the ganglia themselves. This circumstance is easily explained, when we consider that the heart, stomach, and lungs receive their nerves from this quarter; for no other organs depending principally on the sympatheticus, are placed in so close a connexion as these with the central organs.

The author has convinced himself, by means of the microscopical investigation of each individual point of union that the sympathetic nerve joins the anterior roots of the spinal nerves, and strengthens them in their peripheral direction. It is probable that the posterior roots also receive some filaments; it is, however, difficult to decide this point. The physiological importance of this fact led Dr. V. to extend his observations to the mammalia, and he had the satisfaction to find it confirmed in the rat and mole.

It only remains, now, to describe the ganglia; they all possess a bright yellow colour in the frog, and contain three elementary forms of tissue, viz. globules, filaments, and cellular tissue. This has reference to the ganglia of the spinal nerves, as well as to those of the sympatheticus. They are regularly formed, more round than oval, and only once was a globule observed that seemed to indicate a capsular arrangement with flocculent contents. Their diameter is from 0·00120 to 0·00184 inch.

These ganglia present, likewise, here and there, yellow patches, which appear to be colouring matter. Dr. V. has never been able to discover any organization in this, and is inclined, therefore, to consider it to be fluid. In some cases it appeared that the globules themselves contained the colouring matter, which appeared in the form of dots on their surface.

"The filaments in both sorts of ganglia possess the following characters in common: 1. They form bundles in their passage through the ganglia, which are easily torn in a longitudinal direction. They are not easily torn across, but can be pursued as single filaments through a considerable extent. These circumstances seem to indicate that they do not lose their neurilema. 2. I have never found varicose fibres in them, although I have so frequently investigated them. 3. Nor have I ever perceived anastomosis or subdivision of the primitive filaments. 4. If a ganglion be dissected with care, it will be often found that fasciculi of these filaments traverse it, entering from a nervous branch on the one side, and passing out on the other into a different one. In cases where a ganglion stands connected with many branches, an individual branch may be found entering on one side, and, upon leaving it, distributing itself to some or all of those remaining. The filaments never terminate in the globules, nor do they pass through but between them; neither have I ever found that the globules are surrounded or inclosed by them; they rather appear to lie between the globules in distinct fasciculi; and, indeed, the proportion of globules is sometimes very inconsiderable, whilst here and there considerable accumulations of globules may be observed, without any filaments running between them. From these irregularities it is questionable whether the globules really consist of nervous matter. These globules are likewise met with in the sympathetic nerves." *Müller's Archiv.* 3 *Heft.* 1838.

---

*Case of Anæsthesia (Loss of Sensation,) in the course of distribution of the Fifth Nerve, with Remarks.* By Dr. Romberg.

[This paper is a valuable contribution to the diagnosis of nervous diseases. Its author has for many years been occupied with similar investigations; and those who have studied at the University of Berlin know how to estimate the industry and affability which so eminently characterize him. If we are not misinformed, Dr. R. has recently been elevated to the professorial chair, so that it is to be hoped that the extended sphere now opened to him will not be without good effects.]

It is well known how few have been the instances of pathological conditions of the fifth nerve which have been recorded, compared with the numberless cases of affections of the N. facialis, since Sir C. Bell's work has given to this branch of pathology so much interest. It must be admitted, however, that diseases of the fifth pair furnish more satisfactory evidence of the real nature of their function than can be obtained by means of vivisections. The following is a case of interest, both with regard to the physiological character of this nerve and the diagnosis of its abnormal conditions:

A widow, aged forty-two, fell down stairs backwards, and received a violent blow on the occiput. A twelvemonth afterwards her catamenia ceased. From this time she was subject to attacks of sneezing, which increased so much, both in violence and duration, that the slightest circumstance provoked the convulsions, and her sleep was continually disturbed by them. The examination of the nostrils afforded no clue to the cause of this condition, and I, therefore, sought to account for it in the injury done to the head, implicating, possibly, the nasal filaments of the fifth nerve. On investigating the regions to which the first and second branches of the fifth are distributed, I found the sensibility undiminished, but upon arriving at the region of the third branch, I found complete anæsthesia in its whole extent. I will here detail the symptoms, as I have repeatedly demonstrated them to my pupils, and likewise in the presence of Prof. Müller and other friends. I always took the precaution of effectually binding the patient's eyes, which I deem to be necessary in such investigations, in order to guard as much against simulation as to prevent the patient being misled by the sight of the instrument used.

The left half of the under lip, both on its external and internal surface, and the left half of the chin did not betray the least sensibility on being pricked with a sharp vaccinating needle; this was likewise the case with the left ear and the meatus externus. The same parts were insensible to the flame of a taper. The insensibility extended upwards to the left temple, bordering on the hairy scalp, and including the tongue also. There was no pain caused by pricking, nor was there perception of heat or cold on the side, point, or surface of the tongue on this side.

On the right side of the head all these parts were in full possession of their sensibility, and indeed all the other sensitive nerves of the left side preserved their integrity, so that the limits of the distribution of the third branch of the fifth could be accurately marked out by means of pricking the skin. If the needle came in contact with the skin of the temples towards the forehead, the patient gave immediate signs of pain, from the presence of branches of the frontalis: the same result ensued on injuring the integuments covering the horizontal portion of the lower jaw, from the presence of the subcutaneous branches of the third cervical nerve.

Besides the loss of sensation, with reference both to heat and cold and mechanical injury, which the tongue displayed, I found that the sense of taste was obliterated. No kind of substance, fluid or solid, bitter or otherwise, produced the least impression on the left side, whilst on the right, they were discriminated with precision. I tried this experiment with various substances, as colocynth, various salts, acids, &c.

Notwithstanding this partial disturbance of the sensibility of the left side of the face, nothing of the sort existed in the motory function. Neither the expressive or mimical (*mimische*), nor the respiratory or masticatory motions were impaired. The same was the case with the masticatory and articulatory motions of the tongue. The nutrition of the left side was unimpaired; the dimensions, colour, and moisture of both sides being alike, and blood flowing with the same readiness, and ceasing equally soon on the left side as on the right.

From these premises I drew the following diagnosis: "The anæsthesia which extends throughout the distribution of the third branch of the portio major of the fifth, indicates an isolated affection of this nerve, and, indeed, a compression of its trunk, since the loss of sensibility has been unaccompanied throughout with painful sensation in the parts to which the nerve is distributed. The cause of the existing pressure must include the whole of the primitive filaments belonging to this subdivision of the nerve, and, consequently (in all probability), the trunk of the nerve itself, for there is an entire absence of sensibility in all the organs supplied by it; and, on the other hand, the immunity of the parts supplied by the remaining two branches of the fifth, prove that the pressure cannot be situated in the Gasserian ganglion, else there would be more or less implication of other parts in the anæsthesia; neither can the seat of pressure be external to the foramen ovale of the sphenoid bone; for from this point the motor and sensitive fibres of the third branch are in such close juxtaposition, that it would be impossible that one class of functions should be so completely annihilated and the other be left unimpaired, (it will be remembered that the masticatory motion of the left side was not weakened.) I am, therefore, led to assume the presence of a tumour, either of the dura mater or of the bone, so situated as to cause pressure on the third branch of the fifth nerve, previous to its passage out of the foramen ovale."

On the 19th March, the patient died of dropsy, and the body was brought for examination to the Anatomical Theatre in Berlin. Previous to the section, I repeated my diagnosis to those present, viz., Prof. Müller and Drs. Henle, Schwann, and Philipp.

The investigation of the contents of the cranium gave the following result: The surface of the brain was covered with a gelatinous, and more or less white, opaque exudation. A portion of the brain, of about the size of a walnut, situated on the inferior surface of the posterior lobe of the left hemisphere, and corresponding to the posterior horn of the lateral ventricle, was softened, but without any sign of vascular injection in the neighbourhood. In other respects both the brain and spinal cord were normal. The third branch of the fifth nerve, just at its point of entering the foramen ovale, was surrounded by a reddish vascular tissue, consisting partly of fibres and vesicles. Closer investigation showed it to be an exudation into or hypertrophy (Wucherung) of the neurilema, passing into the dura mater, in the direction of the origin of the nerve, and becoming gradually lost upon the neurilema, inclosing the nerve after its passage through the foramen ovale. The neurilema was thickened, and reddish throughout that portion which lies in the sphenoid bone, but becoming less so as it approached the spot where the ganglion oticum lies in contact with it. As far as the neurilema was altered in appearance, the nerve appeared to be thicker and firmer, and to possess a slight yellow colour. The third branch of the Gasserian ganglion was the only portion of the nerve that had suffered any alteration. The motor branch lay to the inside of the nerve, and joined it below the spot described. All the nerves supplying the buccinator, pterygoid, and temporal muscles were normal, as likewise those of the tongue and lower jaw, the quintus of the right side, and the glosso-pharyngeal nerve of both sides.

Remarks. These observations could not have been made at a more opportune season than the present, when the controversy concerning the gustatory nerve engages so much the attention of physiologists. I think I shall not be presuming too much, if I consider this case as conclusive evidence of the truth of the theory which maintains that real gustatory fibres are at least contained within this branch.

I express myself thus in order to avoid the general but erroneous view, that this nerve is an aggregate of homogeneous filaments; for this case incontestibly proves that sensitive and gustatory elements are included in it. Pathological facts are in this case of infinitely more value than experiments on animals; and this is true of all the perceptions of sense. In the experiments respecting the participation of the glosso-pharyngeus and lingualis in the sense of taste, neglect of this consideration led to erroneous conclusions. The sensitive functions of the tongue were limited to those of mere sensation and taste; and a third, one of considerable importance, was overlooked, although it is one that the most simple experiment may serve to establish. For instance, if the finger be passed over the point, edge, or middle of the tongue, nothing but ordinary sensation is excited; but as soon as we approach the papillæ vallatæ and the root of the tongue, the feeling of nausea is excited together with a sense of choking, a decided reflex action. It is in these papillæ, and also in the velum and other parts which when irritated give rise to the same sensations, that the filaments of the nervus glosso-pharyngeus* are distributed. In most of the experiments made upon animals with reference to this circumstance,† it is stated that nausea and strangulation

* The author terms the nerves frequently "*via*," thus, "via glosso-pharyngea" instead of nervus glosso-pharyngeus. His object is to avoid the apparent confounding several distinct kinds of filaments together, under a term which implies uniformity.—Trans.

† See Panizza and Valentin; Repert. für Anatomie u. Physiol. 1837. 2 Bd. 2 Abthl. S. 220.

were excited in cases where the glosso-pharyngeus was uninjured; but I think it was incorrect to ascribe their production to the influence of taste. I consider that the greater abundance of the papillæ vallatæ has reference, in animals, to the instinct possessed by them, inasmuch as I consider the sensation of nausea, &c., to be of more importance in enabling them to discriminate between noxious and innocuous food than mere taste. The observations of Rudolph Wagner* seem to strengthen this opinion, for he shows that papillæ vallatæ appear to have constant reference to the alimentary instinct of the different mammalia in their form, number, size, and situation. I need not observe that I differ from him in attributing the function of taste to the glosso-pharyngeus.

From the reasons above stated, I do not hesitate in terming the glosso-pharyngeus *the nerve of alimentary instinct*. This, too, explains its uniform presence in all classes of animals, whilst the lingualis is wanting in birds.

Besides the specific sensitive function of the glosso-pharyngeus, its peculiar reflex action, above alluded to, is very deserving of attention. It is exceedingly interesting to observe how different are the reflex functions peculiar to contiguous organs, even when produced by the same stimulus. The irritation of the vagus in the glottis produces cough; in the fauces, the same nerve being irritated, excites the act of swallowing; the glosso-pharyngeus causes the phenomena we have described. A very instructive instance of this variety of reflex action is given by Marshall Hall, (Lectures, &c. p. 23,) where a person introduced a feather into the mouth in order to cause vomiting, by irritating the fauces, but passing it too far it became subject to the peculiar action of the œsophagus, and was drawn into the stomach, These reflex actions may be made use of as reagents in the investigation of the offices of sensible nerves; and as I lately found, whilst experimenting upon a horse, that irritation of the nervus vagus in the neck caused cough, so the irritation of the glosso-pharyngeus would, I doubt not, in all cases produce the sensation of choking.

The next important commentary that this case affords is the elucidation of the law of *isolated conduction* (isolirte Leitung) and *co-sensation*. The first of these laws ensured the accuracy of the diagnosis, whilst the latter offers an explanation of the convulsive sneezing. For we can best comprehend the connexion of the cause and effect, by supposing that this peculiar convulsive sneezing was produced through the radiation of the sensation, and transference of the irritation of the filaments of the third branch, to the nasal filaments of the first, (whether in the ganglion Gasseri or within the central organ I will not decide,) and by means of the reflex respiratory motion. The co-sensation in the cerebral end of the nasal twigs was so acute and the tension so considerable, that any the least stimulus gave rise to the sternutatory action, so that my experiments on the sensation of the face was often interrupted thereby.

We have hitherto only considered the case in its physiological characters, but it teems likewise in points of pathological interest; particularly of course with reference to the fifth pair of nerves, concerning which we know as yet so little.

In the hyperæsthesia of this nerve (tic douloureux), the confusion and unintelligibility of the writings on the subject afford sufficiently convincing proof how little is known respecting it, and the paralysis of the fifth was a complete terra incognita, first discovered by the genius of Bell. Since that time some few cases have been published, which serve to throw a brighter light upon the subject. The paralysis affects either the sensible or motor branches, or both at once. Simple means suffice to establish this. Further, it must have either a peripheral or central origin; and here I would observe, that the former has a more extended sphere than is usually ascribed to it. It has been a common error in pathology to view the aggregate of nervous fibres, as they leave the basis of the brain as the nervous root or origin of the nerve, and, consequently, to include their diseases, whilst in this part of their course, under the head of affections of the central organs. But a nerve must be considered as peripheral in every portion of it, from its earliest

* Neue Notizen aus dem Gebiete der Natur u. Heilk. No. 75.

origin to its remote termination. Thus, the paralysis of the fifth is peripheral, whether its inducing cause be seated in the surface of the face, in the sphenoid bone, the ganglion Gasseri, or in the neighbourhood of the pons varolii. The real locality of the affected portion may be determined diagnostically. The more isolated the anæsthesia, the more peripheral is the cause.

Thus, when caused by the extraction of a molar tooth, the anæsthesia was confined to half of the lower lip, as in other cases it is limited to the alæ nasi or the surface of the eye, &c. We may thus pursue diseases of the nerve in its course, until the loss of sensibility of the entire surface of the face supplied by it, combined with paralysis of the masticatory muscles, indicates an affection either of the ganglion Gasseri or the parts in its immediate neighbourhood. In cases where the ganglion is affected, another class of symptoms makes its appearance, possessing considerable physiological interest, viz., disturbance of the vegetative functions in the parts which are at the same time deprived of their sensation; this produces in the eye inflammation, suppuration, and ulceration; in the nasal and oral cavities redness, and hemorrhage, and wasting of the gums. Such instances have been observed by Serres and Abercrombie.*

*Müller's Archiv.* 3 Heft. 1838.

---

*On Transfusion of Blood.* By Professor Th. Bischoff, of Heidelberg.

Dr. B. communicates some very interesting remarks on this subject. He had formerly published his confirmation of the experience of Prévost, Dumas, and Dieffenbach, to the effect that fresh and unagitated blood procured from any of the mammalia, caused instantaneous death upon being injected into the veins of a bird. Upon repeating this experiment in his class, in the course of this and the previous summer, he was much astonished at not finding the usual fatal result to occur. In vain did he attempt to explain the cause of the disappointment, until it occurred to him whether, perhaps, there may be a difference in the effect of venous and arterial blood. In his former and latter experiments, he had taken a cat, rabbit, or young dog, and obtained blood from it by cutting its throat. It was therefore possible, that at different times the syringe had taken up different kinds of blood. To decide this, the following experiments were performed: In one leg of a dog the vena cruralis was exposed, and the arteria cruralis in the other. First, some blood was taken from the vein, and about a drachm was injected into the left jugular vein of a healthy cock. The bird died in a few seconds, under the most violent convulsions. A portion of blood from the artery of the other leg was then injected into the corresponding vein of a hen. The bird was powerfully affected by it, but the experiment did not prove fatal; in a short time it had recovered itself, and a small quantity of venous blood was injected, upon which the fowl died immediately. Precisely similar experiments were tried some time afterwards, and with the same results; here a few drops only of the venous blood proved fatal. A strong goose, also, bore the injection of arterial blood, but venous blood caused the most violent convulsions and death. What could be the cause of this remarkable difference? In order to avoid the possibility of other causes operating, every precaution was adopted. Thus, in order to prevent the possibility of plethora, a small quantity of blood was allowed to escape first; due care was also taken to avoid the injection of air into the veins, which however is by no means always attended with fatal consequences. There is then no doubt left, in the opinion of Dr. B., that it is the venous blood which exercises the fatal influence; how it acts, is a question which remains to be solved; and its solution will no doubt prove of service in increasing our knowledge of the differences between venous and arterial blood. *Müller's Archiv.* 4 *Heft.* 1838.

* Diseases of the Brain, p. 424. Last Edition.

*On the Temperature of the Vagina and Uterus before and during Menstruation, and of the Vagina during Pregnancy.* By J. C. G. FRICKE, Hamburg.

THIS paper contains an account of thirty-four experiments made on twenty-four women. The thermometer (Reaumur's) used in examining the vagina was bent at a right angle, and introduced as deep as possible into the vagina, the labia being closed on it; that for the uterus had a very fine and longish bulb, which was introduced from four lines to half an inch into the uterus. The speculum employed was previously warmed. The experiments were made between ten and eleven, A.M. The following conclusions are deducible from the whole observations: 1. That the temperature of the external air affects the axilla, but not the internal parts. 2. That the vagina is always warmer than the axilla and uterus; but that the uterus is warmer than the axilla. 3. That both menstruation and pregnancy have little or no effect on the temperature of the vagina.

*Zeitschrift, f. d. g. H. Hamburg, Nov.* 1838.

